# The extent of extranodal extension as a prognostic indicator in papillary thyroid cancer

**DOI:** 10.20945/2359-4292-2025-0094

**Published:** 2025-10-23

**Authors:** Michelle Azevedo Gomes, Mirian Carvalho de Souza, Mário Lúcio Cordeiro Araújo Júnior, Fernanda Vaisman, Sérgio Ricardo Carvalho de Araújo, Priscila Valverde Fernandes, Fernando Luiz Dias

**Affiliations:** 1 Departamento de Cabeça e Pescoço, Instituto Nacional de Câncer, Rio de Janeiro, RJ, Brasil; 2 Instituto Nacional de Câncer, Rio de Janeiro, RJ, Brasil; 3 Departamento de Patologia, Instituto Nacional de Câncer, Rio de Janeiro, RJ, Brasil; 4 Departamento de Endocrinologia, Instituto Nacional de Câncer, Rio de Janeiro, RJ, Brasil; 5 Departamento de Pesquisa Populacional, Instituto Nacional de Câncer, Rio de Janeiro, RJ, Brasil

**Keywords:** Papillary thyroid cancer, extranodal extension, prognosis, metastasis

## Abstract

**Objective:**

Extranodal extension (ENE) is acknowledged as a significant prognostic factor associated with recurrence, distant metastasis, and reduced disease-specific survival in patients with papillary thyroid carcinoma. However, the impact of the extent of extranodal extension on the clinical outcomes of these patients remains insufficiently understood. This study aimed to estimate the risk of detecting distant metastasis in patients with varying degrees of ENE according to a novel stratification method.

**Materials and methods:**

This retrospective study utilizes medical records and slide reviews of papillary thyroid cancer patients who underwent therapeutic neck dissection. A new stratification system was developed, based on the circumferential rupture of the lymph node capsule. It is defined as Focal ENE when less than one-third of the lymph node capsule is ruptured and as Diffuse ENE when one-third or more of the capsule is involved.

**Results:**

Eighty-nine patients participated in the study, with 19% diagnosed with distant metastasis within a 96-month follow-up period. The presence of diffuse extranodal extension was associated with a risk approximately six times higher than in patients without ENE for the detection of distant metastasis at 96 months, after adjustment for age group (HR = 6.41; 95% CI: 1.7-23.8; p = 0.006).

**Conclusion:**

A greater extent of extranodal extension is linked to a heightened risk of detecting distant metastasis and should thus be considered in the therapeutic decision-making process.

## INTRODUCTION

The incidence of thyroid cancer has been increasing in recent decades, primarily due to a rise in well-differentiated thyroid carcinoma cases (^1^). These carcinomas constitute approximately 90% of new thyroid cancer cases in both men and women, with most being of the papillary subtype (^2^). Papillary thyroid carcinomas are generally slow-growing tumors and have the best survival rates among all types of thyroid cancer (^3^). However, some patients experience challenging clinical outcomes, making the identification of prognostic factors for these patients a subject of study in recent decades (^4^-^9^).

While certain prognostic factors, such as being aged ≥ 54 years, larger tumor size, extrathyroidal extension, angiolymphatic invasion, and aggressive histological variants, are well-known, controversies persist regarding the significance of some features of regional metastases, such as the number of positive lymph nodes, size of affected lymph nodes, and the presence of extranodal extension (^5^).

Extranodal extension refers to the invasion of tumor cells beyond the capsule of a lymph node into the surrounding soft tissue (^10^). The current classification of extranodal extension for head and neck tumors, as well as other cancers, is divided into microscopic ENE, where the tumor extends beyond the lymph node capsule by up to 2 mm, and macroscopic ENE, where the invasion exceeds 2 mm, as adopted by several authors (^11^). Recently, new histopathological classifications of the degree of extranodal extension have been proposed, based on the specific characteristics of the tumor understudy (^12^,^13^).

The risk stratification system validated by the American Thyroid Association recognizes three lymph node criteria as predictors of recurrence risk: the number of positive lymph nodes, the size of the largest affected lymph node, and the number of lymph nodes with extranodal extension (^14^). However, the American Thyroid Association classification system does not subdivide extranodal extension into microscopic and macroscopic categories, as adopted by some authors (^15^,^16^).

Conversely, the American Joint Committee on Cancer (AJCC) staging system considers only the presence of metastasis in the central (N1a) or lateral compartments (N1b) for the prognosis of patients with papillary thyroid carcinoma. This contrasts with the staging system for head and neck squamous cell carcinoma, where lymph node size, site of nodal disease, and extranodal extension are considered, with the latter being incorporated in its latest edition (^17^).

In papillary thyroid carcinoma, the presence of extranodal extension is known to be associated with an increased risk of metastasis, recurrence, and disease-specific survival (^18^,^19^). The presence of more than three lymph nodes with extranodal extension is associated with recurrence in this type of cancer (^4^).

Extranodal extension visible to the naked eye has been shown to decrease survival in patients with papillary thyroid carcinoma (^16^). However, the relationship between the extent of microscopic extranodal extension and its impact on the prognosis of patients with papillary thyroid carcinoma remains uncertain.

This study evaluated whether a greater extent of microscopic extranodal extension in papillary thyroid carcinoma is associated with a higher risk of detecting distant metastasis. Additionally, a classification of extranodal extension focused on the specific characteristics of papillary thyroid carcinoma is proposed, aiming to improve prognostic stratification.

## MATERIALS AND METHODS

**Figure 1:**
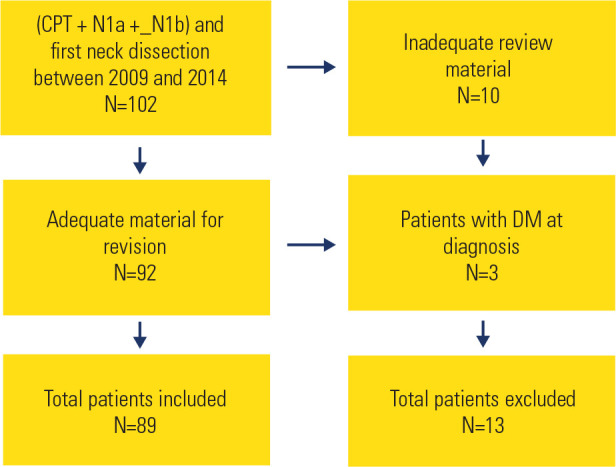
Flowchart of the sample. Out of the 102 patients who underwent surgical interventions during the specified period, 13 were excludedª.

Note: ªCPT = papillary thyroid carcinoma; DM = distant metastasis.

This observational cohort study, which involves retrospective data collection and the review of histopathological slides, included patients aged 18 years or older with papillary thyroid carcinoma. These patients underwent therapeutic cervical lymph node dissection at the National Cancer Institute (INCA) in Rio de Janeiro between 2009 and 2014 (**Figure 1**). The profile of patients at our institution comprises both treatment-naïve individuals and those who underwent previous surgeries elsewhere. Candidates for neck dissection at our institution are patients with suspicious cervical lymph nodes identified clinically or through imaging examinations, confirmed by FNAB or intraoperative frozen section analysis. Patients with distant metastases prior to lymph node dissection or slides unsuitable for review were excluded from the study.

Prognostic data were collected independently of the slide review, and the pathologist was blinded to the outcomes of the patients whose slides were reviewed. Patients were followed for eight years from the date of the first cervical lymph node dissection. Distant metastases detected within the eight-year follow-up were considered, while those occurring beyond this period were censored. The presence of distant metastasis was evaluated using positron emission tomography with 18F-fluorodeoxyglucose, computed tomography, magnetic resonance imaging, or whole-body scans. Imaging tests were requested only when lung metastases were suspected during follow-up.

The tissue blocks and slides were stained previously with Hematoxylin-Eosin. The criteria from the latest edition of the AJCC were used to define extranodal extension, referring to the penetration of tumor cells beyond the lymph node capsule into the perinodal soft tissue. Extranodal extension was classified by the following system proposed by the authors: Focal ENE - less than one-third of the lymph node capsule was ruptured; Diffuse ENE - one-third or more of the lymph node capsule was ruptured. Patients with two or more lymph nodes showing extranodal extension were classified according to the lymph node with the greatest extent of extranodal extension.

Clinical, therapeutic, and pathological characteristics of the study population were described using tables with absolute and relative frequencies. Differences in categorical variables were assessed with Fisher’s exact test.

To estimate the cumulative incidence of distant metastasis diagnosis at eight years, the non-parametric Kaplan-Meier product estimator was used. The initial event was the first therapeutic neck dissection performed at INCA for papillary thyroid carcinoma treatment, while the final event was the diagnosis of distant metastasis or the end of follow-up for those who did not experience this outcome during the eight-year follow-up.

Variables with a *p* < 0.05 in the log-rank test were initially considered for inclusion in the Cox models with a single covariate. In creating the multivariate Cox model, the authors assessed collinearity among candidate variables, retaining only those deemed clinically relevant and not highly correlated. Specifically, ENE degree, the number of lymph nodes with ENE, and the presence/absence of ENE were highly correlated variables; therefore, only the degree of ENE was included in the final model. Likewise, the variable “angiolymphatic invasion” showed a significant association in the single covariate analysis, but with a paradoxical direction of effect (patients without invasion having a higher risk of distant metastasis). Given the limited number of events and the lack of biological plausibility, this result was interpreted as potentially due to chance (type I error), and angiolymphatic invasion was not included in the multivariate Cox model. Thus, the final model was adjusted for age and the degree of ENE, the variables with the strongest clinical plausibility and statistical stability. Data consistency and statistical analysis were performed using Stata 15 software.

This study adhered to the guidelines of Resolution nº 466/2012 of the Brazilian National Health Council and was approved the Research Ethics Committee of INCA. Study number 517.819 was approved on January 29, 2014. The research was conducted according to ethical principles, ensuring patient confidentiality and privacy throughout the study.

### New proposed classification system for extranodal extension

Considering the biological characteristics of cervical metastases in papillary thyroid carcinoma, where even small metastases in the central compartment can exhibit ENE, we propose a new classification system. This system is based on the circumferential rupture of the lymph node capsule and its proportionality relative to the lymph node, rather than solely measuring tumor extension into the perinodal fat.

**Figure 2:**
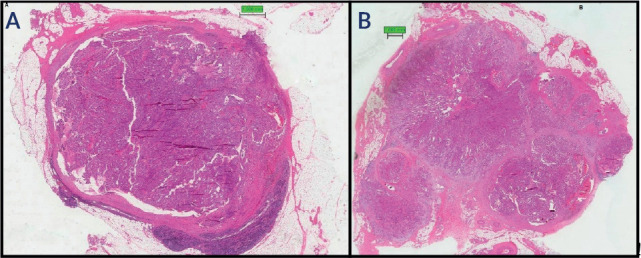
Lymph node metastasis in papillary thyroid carcinoma (H&E, x40). A metastatic tumor rupturing the lymph node capsule and extending into the perinodal fat is shown focally (less than one-third in and diffusely (more than one-third) in **B**.

ENE was classified as focal when the tumor ruptured the lymph node capsule by less than one-third of its circumference (**Figure 2A**), and as diffuse when the rupture extended to more than one-third of its circumference (**Figure 2B**).

This assessment better reflects the unique biological behavior of papillary thyroid carcinoma when compared to the current classification proposed by the AJCC. The AJCC definition considers macroscopic extranodal extension when the lymph node capsule is ruptured and there is tumor invasion into the perinodal fat of more than 2 mm, regardless of the extent of the lymph node capsule rupture.

## RESULTS

### Clinical, therapeutic, and histopathological characteristics of patients with papillary thyroid carcinoma

Of the 89 patients included in the study, 17 developed distant metastasis within 96 months of follow-up. Among these 17 patients, all had pulmonary metastasis, and one-fourth exhibited metastasis to more than one site. The average time to metastasis occurrence was approximately 33 months. About 75% of the patients were female and under the age of 54. Most patients (71%) who were diagnosed with metastases had tumors larger than 4 cm, and more than half were classified as T3 and T4 according to the AJCC 8th edition staging. However, it should be noted that data was missing for more than 25% of the patients since many underwent their initial surgery at an institution other than the one where this study was conducted (**Table 1**).

**Table 1:** Distribution of patients with papillary thyroid carcinoma undergoing therapeutic cervical lymph node dissection, by development of distant metastasis within eight years, and according to clinical, therapeutic, and histopathological characteristics

Characteristics	Distant metastasis		p	Total N (%)
	Yes N (%)	No N (%)		
Total	17 (19.10)	72 (80.90)	NA	89 (100)
Sex				
Male	6 (35.2)	16 (32.7)	0.261	22 (24.8)
Female	11 (64.8)	56 (67.3)		67 (75.2)
Age (years)				
≤54	7 (41.2)	62 (86.1)	**<0.001**	69 (77.5)
≥55	10 (58.8)	10 (13.9)		20 (22.5)
Previous surgery (^2^)				
None	6 (37.5)	52 (73.2)	**0.001**	58 (66.7)
PT	2 (12.5)	5 (7.0)		7 (8.0)
TT	2 (12.5)	11 (15.5)		13 (15.0)
TT+ CLND	6 (37.5)	3 (4.3)		9 (10.3)
RiT before CLND				
Yes	11 (15.3)	4(23.5)	0.474	15(16.8)
No	61 (84.7)	13(73.5)		74(83.2)
Histologic type (^2^)				
Classic	14 (82.3)	59 (84.3)	0.548	73 (^16^)
Others	3 (17.7)	11 (15.7)		14 (84)
Tumor size - cm (^24^)				
<1	0 (0.0)	7 (12.7)	**0.002**	36 (52.9)
1-4	3 (30.0)	40 (72.8)		43(23.0)
>4	7 (70.0)	8 (14.5)		15 (66.3)
EET (^5^)				
Absent	9 (64.3)	57 (81.4)	0.167	66 (78.6)
Present	5 (35.7)	13 (18.6)		18 (21.4)
Angiolymphatic invasion				
Present	8 (57.1)	58 (82.8)	0.062	66 (78.6)
Absent	6 (42.9)	12 (17.2)		18 (27.4)
Margins evaluation (^14^)				
Clear	4 (30.8)	37 (59.7)	0.071	41(54.6)
Involved	9 (69.2)	25 (40.3)		34(45.7)
N				
N1a	0 (0.0)	18 (25.0)	**0.019**	18 (20.2)
N1b	17 (100.0)	54 (75.0)		71 (79.8)
Number of positive LNs				
1-5	5 (29.4)	37 (51.4)	0.115	44 (49.4)
>6	12 (70.6)	35 (48.6)		47 (52.8)
ENE				
Absent	3 (17.6)	41 (57.0)	**0.006**	44 (49.4)
Present	14 (82.4)	31 (43.0)		45 (50.6)
ENE gradient				
None	3 (17.6)	41 (56.9)	**0.007**	44 (49.5)
Focal	5 (29.5)	15 (20.8)		20 (22.5)
Diffuse	9 (52.9)	16 (22.3)		25 (28.0)
Number of LNs with ENE				
0	3 (17.6)	41 (56.9)	**0.004**	44 (49.5)
1-3	10 (58.8)	26 (36.1)		36 (40.4)
≥4	4 (23.6)	5 (7.0)		9 (10.1)
RiT after CLND				
Yes	10 (58.8)	55 (76.4)	0.142	65 (73.0)
No	7 (41.2)	17 (23.6)		24 (27.0)
Treatment response				
Excellent response	0 (0)	34 (47.2)	**0.000**	34 (38.2)
Biochemical incomplete	1 (5.9)	16 (22.2)		17 (19.1)
Structural incomplete	14 (82.3)	11 (15.3)		25 (28.1)
Undetermined	2 (11.8)	11 (15.3)		13 (14.6)
Subsequent surgery				
Yes	6 (35.3)	11 (15.3)	0.059	17 (19.1)
No	11 (64.7)	61 (84.7)		72 (80.9)

*p*-Values were obtained using Fisher’s exact test. (#) Number of missing data; PT: partial thyroidectomy; TT: total thyroidectomy; CLND: cervical lymph node dissection; Rit: radioiodine therapy; EET: extrathyroidal extension; T: Tumor classification according to the 8th edition of the American Joint Committee on Cancer (AJCC); N: nodal classification according to the 8th edition of the AJCC; LN: lymph nodes; ENE: extranodal extension; NA: not available.

Regarding lymph node sites, all patients who developed distant metastasis had disease in the lateral compartment. Extranodal extension was present in a little over half of the patients (45/89), and among these, most exhibited diffuse extranodal extension (25/45). Among those who developed metastasis, more than 80% had extranodal extension, with a majority presenting diffuse extranodal extension. Approximately 40% of patients had between one and three lymph nodes with extranodal extension, while only 10% had four or more lymph nodes exhibiting the same characteristic. Notably, for patients diagnosed with metastasis, 60% had between one and three lymph nodes affected (**Table 1**).

Concerning therapeutic response, among the patients who detected distant metastasis, more than 80% had structural disease after neck dissection. In contrast, among those without distant metastasis, 47% exhibited an excellent response following neck dissection. In both the M0 and M1 groups, less than half of the patients underwent additional surgical intervention in the neck (**Table 1**).

### The probability of detecting distant metastasis is greater among patients with a higher extent of extranodal extension.

**Figure 3:**
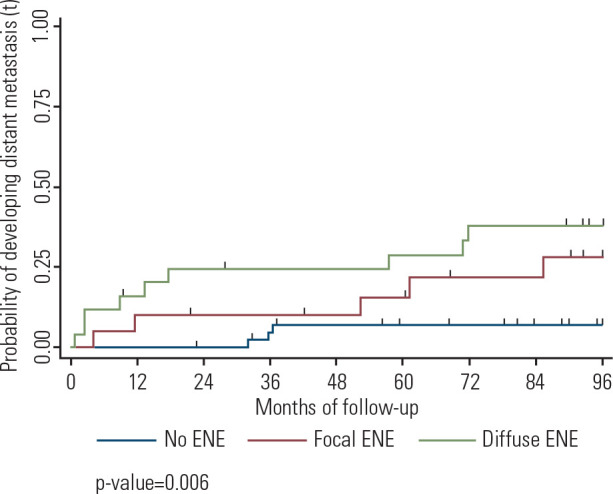
Estimated probabilities of detecting distant metastasis related to the extent of extranodal extension in patients with papillary thyroid carcinoma undergoing therapeutic cervical lymph node dissection.

The overall probability of detecting metastasis within 96 months was 20.4%. While patients without extranodal extension had a 7.1% probability (95% CI, 2.3-20.4) of detecting distant metastasis within eight years, those with focal extranodal extension had a 28.2% probability (95% CI, 12.7-55.5). For patients with diffuse extranodal extension, the probability was 37.7% (95% CI, 21.6-60.2) (*p* = 0.006) (Supplementary **Table 1**). In summary, a greater extent of extranodal extension is associated with an increased probability of detecting distant metastasis within eight years (**Figure 3**).

The presence of a higher degree of ENE represents a sixfold greater risk of diagnosing distant metastasis compared to patients without ENE. In the analysis of single covariate Cox models, it was observed that in patients aged 55 years and above, the presence of extranodal extension, more than three lymph nodes with extranodal extension, and a higher extent of extranodal extension conferred a greater risk of diagnosing distant metastasis (**Table 2**).

**Table 2:** Factors influencing the likelihood of detecting distant metastasis within 96 months in patients with papillary thyroid carcinoma undergoing therapeutic neck dissection

Variables	Single covariate - Cox model				Multivariate - Cox model		
	HR	95% CI	p		HR	95% CI	p
Age (years)							
≤55	1.00				1.00		
≥55	5.98	2.3-15.7	**<0.001**		6.41	2.4-17.2	**<0.001**
ENE							
Absent	1.00						
Present	5.35	1.5-18.6	**0.008**				
ENE gradient							
None	1.00						
Focal	4.06	0.9-16.9	**0.055**		5.20	1.2-21.9	**0.025**
Diffuse	6.50	1.76-24.0	**0.005**		6.41	1.7-23.8	**0.006**
Number of LNs with ENE							
0	1.00						
1-3	4.43	1.2-16.1	**0.024**				
>3	11.32	2.5-50.9	**0.002**				

ENE: extranodal extension; LN: lymph nodes; HR: hazard ratio; CI: confidence interval.

After evaluating collinearity and robustness of associations, the final multivariate model was adjusted for age and the degree of extranodal extension (Supplementary **Table 2**). Patients with diffuse extranodal extension have a sixfold greater risk of developing distant metastasis within 96 months (95% CI: 1.7-23.8), compared to those without this characteristic. This condition remains significant even after adjustment for age (**Table 2**).

## DISCUSSION

Our findings indicated that patients with a higher degree of extranodal extension have an increased likelihood of detecting distant metastases among those with papillary thyroid carcinoma. A new classification for the extent of extranodal extension, which considers the characteristics of metastases in papillary thyroid carcinoma, has been proposed. Even with small lymph nodes in the central compartment, extranodal extension can still occur. Extranodal extension is termed focal when the tumor breaches the lymph node capsule by less than one-third of its circumference (**Figure 1A**), and “diffuse” when the rupture extends to more than one-third of its circumference (**Figure 1B**). The presence of diffuse extranodal extension indicates a worse prognosis in patients with papillary thyroid carcinoma.

Approximately 19% of the patients in this study presented with distant metastasis. Previous studies have shown that this percentage varies between 2%-10% (^20^-^22^). However, this discrepancy between our findings and those of other studies may be attributed to our study population, which consisted of intermediateand high-risk patients, unlike other studies that included patients with a lower likelihood of detecting distant metastasis.

The patients who presented with extranodal extension represented just over half of the study population. These results are analogous to those presented by Kim and cols. (^23^) in a retrospective study conducted at Samsung Medical Center in Korea, which found extranodal extension in 193 out of 369 patients (52%) with papillary thyroid carcinoma and cervical nodal disease (^23^).

This proportion is higher than that found by other authors (^19^). This difference may be attributed to the evolving definition of extranodal extension, which has undergone numerous changes over the years and has been standardized more recently (^10^,^24^,^25^). This new definition was used in our study, as well as in the study by Kim and cols. (^23^), with similar results.

All patients who presented distant metastasis had positive lymph nodes in the lateral compartment, and none of those with lymph nodes exclusively in the central compartment developed distant metastasis. In a meta-analysis by Vuong and cols. (^22^), three studies comprising 1,072 patients with papillary thyroid carcinoma investigated the relationship between the presence of positive lymph nodes in the central compartment and distant metastasis. The results showed that patients with central compartment lymph nodes did not present a significant risk for distant metastasis (OR = 0.82; 95% CI 0.25-2.72). Conversely, patients with lateral lymph nodes had a significantly higher risk of distant metastasis (OR = 5.75, 95% CI 2.91-11.36), thus corroborating the findings that nodal disease in the central compartment does not significantly impact the prognosis of patients with papillary thyroid carcinoma as it does in the lateral cervical compartment.

The presence of extranodal extension represented a higher risk of detecting distant metastasis among the patients in the study, as evidenced by the difference between the curves of patients with and without extranodal extension (**Figure 2**). Sugitani and cols. (^26^) were the first authors to describe the close relationship between distant metastasis in papillary thyroid carcinoma and extranodal extension, observing its presence as a common feature in many patients who developed distant metastasis. Jeon and cols. (^27^) reviewed a series of 8,808 patients and found an association between the presence of extranodal extension and distant metastasis even in papillary thyroid microcarcinomas (OR = 5.50; 95% CI 1.08-33.64; *p* = 0.045). In addition to its influence on the detection of distant metastasis, extranodal extension also impacts disease-specific survival and recurrence in patients with this characteristic (^19^). Therefore, it is recommended to consider extranodal extension in the histopathological examination of patients with thyroid cancer, and this factor should be included in future staging systems.

The present study identified a higher likelihood of patients detecting distant metastasis in relation to the extent of extranodal extension, presenting a sixfold increased risk of distant metastasis for patients with a greater extent of extranodal extension, regardless of age.

In the series by Ito and cols. (^15^), patients with extranodal extension were categorized into those with the extension reaching adjacent organs and those with metastatic nodules completely invading adjacent organs. The authors did not find statistically significant differences in the prognosis of these two groups. However, it is likely that the absence of differences in patient outcomes based on Ito and cols.’ criteria is due to the use of only macroscopic criteria to define extranodal extension, excluding patients who exhibited this characteristic solely on a microscopic level, as was performed in our cohort.

Among the limitations of this study are the limited number of patients facing a rare outcome and the lack of comparability with existing classifications. Given the relatively small sample size of 89 patients, the statistical power of the study may be limited, necessitating cautious interpretation of the results. An important aspect concerns the selection of variables for the multivariate model. Although angiolymphatic invasion showed a statistically significant association in the univariate analysis, the effect was paradoxical, with patients without angiolymphatic invasion exhibiting a higher risk of distant metastasis. We interpreted this as a spurious finding, likely related to the limited number of events and a type I error; hence, angiolymphatic invasion was not retained in the final model. Additionally, the degree of ENE and the number of lymph nodes with ENE are strongly correlated variables. To avoid multicollinearity and unstable estimates, we opted to include only the degree of ENE in the final analysis. This strategy aimed to enhance the reliability and interpretability of the results. Furthermore, the slides were analyzed by only one pathologist, although this pathologist has decades of experience with this type of cancer.

Due to the retrospective nature of our study, patients did not undergo standardized imaging workups to screen for distant metastases prior to surgery. Consequently, the documented date of diagnosis corresponds to the time of detection and does not exclude the possibility that metastases were present at the time of surgery but remained undiagnosed. Although the indolent behavior of the CPT corroborates the hypothesis that distant metastasis occurs after spreading to the neck, this analysis should be approached with caution.

To our knowledge, this study is the first to propose a classification of microscopic extranodal extension that considers the specific characteristics of papillary thyroid carcinoma metastases for patients’ prognosis.

Future studies with larger patient populations that compare our newly proposed classification with existing ones are essential for a better understanding of the impact of the degree of extranodal extension and its applicability. Understanding this tumor feature may also be important to define the appropriate follow-up time for patients. It may also assist in better determining the indication and dose of radioiodine therapy for intermediate-risk patients. Clinical studies are needed to assess whether therapeutic changes could influence the development of distant metastasis.

## Data Availability

datasets related to this article will be available upon request to the corresponding author.

## SUPPLEMENTARY MATERIAL

**Table 1:** Probability of developing distant metastasis within 96 months in patients with papillary thyroid carcinoma undergoing therapeutic cervical lymph node dissection, according to clinical, therapeutic, and histopathological characteristics

Characteristics	Probability of developing DM		log-rank *p*
	PM96	95% CI	
Total	20.42	(13.2-30.9)	NA
Sex			
Male	30.0	(14.6-55.3)	0.286
Female	17.3	(10.0-29.2)	
Age - years			
≤54	10.6	(5.2-21.0)	**<0.001**
≥55	53.8	(33.4-77.0)	
RiT before CLND			
Yes	27.3	(11.2-57.5)	0.588
No	18.6	(11.3-30.0)	
Histologic type (^2^)			
Classic	20.6	(12.7-32.4)	0.8335
Others	22.0	(7.7-54.1)	
Tumor size - cm (^24^)			
<1	-	-	**<0.001**
1-4	7.0	(2.3-20.1)	
>4	50.8	(28.0-78.2)	
EET (^5^)			
Absent	14.6	(7.9-26.3)	0.079
Present	28.9	(13.1-56.2)	
Angiolymphatic invasion			
Present	13.2	(6.8-24.7)	**0.024**
Absent	33.3	(16.6-59.6)	
Margin evaluation (^14^)			
Clear	11.1	(4.3-27.0)	0.051
Involved	-	-	
N			
N1a	-	-	**0.032**
N1b	25.1	(16.5-37.4)	
Number of positive LNs			
1-5	13.4	(5.8-29.5)	0.094
≥6	26.8	(16.2-42.4)	
ENE			
Absent	7.1	(2.3-20.4)	**0.003**
Present	33.5	(21.3-50.1)	
ENE gradient			
None	7.1	(2.3-20.4)	**0.007**
Focal	28.2	(12.7-55.5)	
Diffuse	37.7	(21.6-60.2)	
Number of LNs with ENE			
0	7.1	(2.3-20.4)	**0.001**
1-3	29.6	(17.0-48.1)	
≥4	50.0	(21.5-86.3)	
RiT after CLND			
Yes	15.9	(8.9-27.7)	0.171
No	31.9	(16.6-55.8)	
Treatment response			
Excellent response	-	-	**<0.001**
Biochemical incomplete	6.7	1(38.74)	
Structural incomplete	60.8	(41.5-80.5)	
Undetermined	17.5	(4.7-53.9)	
Subsequent surgery			
Yes	39.2	(19.8-67.4)	0.090
No	15.8	(9.1-26.8)	

When n < 10, the probabilities were not showed.

DM: distant metastasis; EET: extrathyroidal extension; ENE: extranodal extension; LN: lymph nodes; CI: confidence interval; PT: partial thyroidectomy; TT: total thyroidectomy; CLND: central lymph node dissection; NA: not available.

**Table 2:** Single covariate cox proportional hazards model for the risk of developing distant metastases in patients with papillary thyroid carcinoma undergoing therapeutic cervical lymph node dissection

Characteristics	Hazard ratio	95% CI	p
Age - years			
≥54	1		
≤55	5.97	(2.3-15.7)	**0.000**
EET (^5^)			
Absent	1		
Present	2.57	(0.9-7.7)	0.091
Angiolymphatic invasion			
Present	1		
Absent	3.16	(1.1-9.1)	**0.033**
Margins evaluation (^14^)			
Clear	1		
Involved	3.04	(0.9-9.8)	0.064
Number of positive LNs			
1-5	1		
≥6	2.37	(0.83-6.7)	0.104
ENE gradient			
None	1		
Focal	4.06	(0.96-16.9)	0.055
Diffuse	6.50	(1.7-24.1)	**0.005**
Number of LNs with ENE			
0	1		
1-3	4.43	(1.2-16.1)	**0.024**
≥4	11.3	(2.5-50.9)	**0.002**
RiT after CLND			
Yes	1		
No	1.9	(0.7-5.1)	0.179
Subsequent surgery			
Yes	1		
No	0.4	(0.16-1.2)	0.100

EET: extrathyroidal extension; ENE: extranodal extension; LN: lymph nodes; CI: confidence interval; CLND: central lymph node dissection.
